# The protective role of employment in depression: insights from 2005 to 2018 NHANES information

**DOI:** 10.3389/fpsyt.2024.1455122

**Published:** 2024-12-09

**Authors:** Fang Li, Zheng Chu

**Affiliations:** ^1^ Department of Marxism, Xuzhou Medical University, Xuzhou, Jiangsu, China; ^2^ Department of Forensic Medicine, Xuzhou Medical University, Xuzhou, Jiangsu, China; ^3^ Jiangsu Medical Engineering Research Center of Gene Detection, Xuzhou, Jiangsu, China

**Keywords:** depression, employment, sociodemographic factors, poverty-to-income ratio (PIR), NHANES, mental health policy

## Abstract

**Background:**

Depression is closely associated with employment status, which serves as a complex social determinant. This study explores the relationship between employment status and depression.

**Methods:**

Data from the National Health and Nutrition Examination Survey (NHANES) spanning 2005 to 2018 were analyzed. The study included 29,452 participants aged 20 and older with complete data on employment and depression. Logistic regression models were applied to examine the association between employment and depression, adjusting for covariates such as age, gender, ethnicity, marital status, education, poverty-to-income ratio (PIR), BMI, diabetes, hypertension, smoking, and alcohol status.

**Results:**

The study revealed an overall depression prevalence of 8.61%, with significant variation across employment statuses—non-employed (11.77%), part-time (6.58%), and full-time (4.52%) workers. Employed individuals, particularly those in the 40-59 age group and with lower PIRs, showed reduced odds of depression (OR 0.42, 95% CI: 0.36–0.48, p=0.000). Stratified analyses confirmed lower depression odds for employed individuals regardless of gender or marital status.

**Conclusion:**

Employment status has a significant impact on depression risk, with full-time work showing the strongest protective effect against depressive symptoms. The study underscores the role of sociodemographic factors in depression and highlights the need for policies promoting stable employment and mental health support, especially for economically vulnerable groups.

## 1 Introduction

Depression, the foremost cause of disability and poor health globally, imposes a significant burden on individuals, families, and healthcare systems (1). Characterized by persistent sadness, loss of interest, and a decline in cognitive and physical functions, depression disrupts personal well-being, social engagement, and occupational performance (2). The widespread incidence of depression globally highlights the pressing need to comprehend its contributing factors, thereby enabling the development of effective prevention and intervention strategies (3). Among the various factors associated with depression, employment status stands out as an essential, intricate, social determinant (4). Employment is more than a means of livelihood; it constitutes a foundational element of social identity, offers a framework of structure and routine, and serves as a conduit for social support and self-worth. The potential impact of employment on depression is multifaceted, involving economic stability, social integration, and psychosocial well-being (5). However, the relationship between employment and depression is nuanced and can be influenced by a range of individual and contextual factors.

Previous research has shed light on the intricate connections between employment conditions and the prevalence of depressive symptoms. A study focusing on German employees found that current employment status—whether regular full-time, regular part-time, or marginal employment—was associated with depressive symptoms. The research indicated that men experienced elevated depressive symptoms when working regular part-time, while women showed similar symptoms when engaged in marginal employment. These associations were mediated by factors such as job insecurity and leadership quality, highlighting the complex interplay between employment conditions and mental health (6). Besides, the impact of employment status on depression is further underscored in a study examining the role of job autonomy. This research revealed that job autonomy could attenuate the negative effects of depression on employees’ well-being (7). Additionally, the integration of occupational and mental health services is crucial for addressing depression among employees. Individuals with depression in the workplace often face reduced productivity and an increased risk of job loss. Early intervention and a focus on facilitating a return to work can significantly improve outcomes for these individuals (8). Therefore, understanding the influence of employment status on depression is essential for developing effective workplace policies and support systems.

This study leverages data from the National Health and Nutrition Examination Survey (NHANES), a robust and nationally representative dataset that provides a unique opportunity to examine the relationship between employment and depression within a heterogeneous population. NHANES, known for its comprehensive health measurements and detailed sociodemographic information, allows for a sophisticated analysis of the interplay between employment status and mental health (9). The primary aim of this study is to investigate the association between employment status and depression while considering the moderating roles of age, gender, marital status, and economic status as indicated by the poverty-to-income ratio (PIR). By clarifying these relationships, we seek to contribute to the growing body of evidence that informs public health strategies and workplace policies aimed at promoting mental health and well-being. Our findings aim to provide insights that can guide targeted interventions for individuals at risk of depression, particularly in the context of employment and socioeconomic disadvantage.

## 2 Materials and methods

### 2.1 Study population

This study utilized an extensive dataset from NHANES, encompassing data collected across seven cycles from 2005 to 2018. The NHANES program, known for its meticulous health evaluations, engages a team of skilled interviewers to meticulously assess the health and nutritional status of a varied demographic of participants. Operating biennially, NHANES employs a stratified, multistage, and clustered probability sampling methodology. This methodological approach ensures a comprehensive and representative overview of the U.S. population’s health profile. In this study, the inclusion criteria were broadened to include adults who were 20 years of age and older. Participants were included based solely on the availability of complete records concerning their employment status and history of depressive symptoms. Prior to their participation, all individuals provided written informed consent, signifying their understanding and voluntary agreement to contribute to the study. The research, involving human subjects, was conducted with the utmost respect for ethical considerations and was dutifully approved by the National Center for Health Statistics Ethics Review Board. The compiled dataset, a testament to this research endeavor, is readily available for public access on the NHANES website, promoting transparency and further scholarly exploration.

### 2.2 Assessment of employment

The interview conducted for the occupation questionnaire served as the basis for assessing employment. Employment status was determined by using the question “Type of work done last week” and “hours worked last week at all jobs”. These indicators were restructured in accordance with the methodology outlined by Wolfson and Bleich (10), resulting in the classification of employment status into three categories: non-employed (encompassing the unemployed, students, retirees and individuals not actively seeking jobs), part time (1-34 hours per week), and full time (35 hours or more per week).

### 2.3 Assessment of depression

The NHANES employed the Patient Health Questionnaire-9 (PHQ-9) (11) to gauge the intensity of depressive symptoms experienced by participants during a span of two weeks. The PHQ-9 is a validated self-assessment tool comprised of nine questions that correspond to the depressive symptomatology delineated in the American Psychiatric Association’s Diagnostic and Statistical Manual, Fourth Edition. This instrument has established a reputation for its precision and dependability. The PHQ-9 scale assigns a score to each of its nine items on a quartile scale, where 0 signifies “ not at all,” 1 represents “ a few days,” 2 indicates “ more than half of the days,” and 3 denotes “ almost every day.” The overall score is derived by aggregating the individual item scores, yielding a total that spans from 0 to 27. A cumulative PHQ-9 score exceeding 10 was utilized as the threshold for the identification of depressive conditions.

### 2.4 Covariates

Building upon the foundation of existing research and empirical clinical insights, our analysis was executed with meticulous attention to detail, ensuring a comprehensive control of potential confounding factors. We integrated an array of covariates into our multivariable-adjusted models to account for various influences that could skew the results. This suite of variables included, but was not limited to, age (calculated in years), gender, ethnic heritage (classified into distinct categories such as Mexican American, Other Hispanic, Non-Hispanic White, Non-Hispanic Black, and Other Races), levels of educational achievement, the poverty-to-income ratio (PIR), marital circumstances (categorized as Married/Living with partner, Never married, widowed/divorced/separated), body mass index (BMI), alcohol consumption status (present or absent), smoking habits (yes or no), the presence of hypertension (yes or no), and whether individuals had diabetes (yes or no).

Age is classified into three categories: 20-39 years old, 40-59 years old, and over 60 years old. The PIR, which ranges from 0 to 5, was also divided into tertiles for analysis (the lowest tertile T1 ranging from 0 to 1.3; the middle tertile from 1.3 to 3.5; and the highest tertile from 3.5 to 5). Educational attainment was grouped into three categories, with ‘high school grade/GED or equivalent’ serving as the threshold. The assessment of alcohol status was based on the question “Had at least 12 alcohol drinks/1 year?” for 2005-2016 cycles, and question “Ever had a drink of any kind of alcohol?” for the 2017-2018 cycle. Smoking status was determined by asking participants if they had smoked at least 100 cigarettes in their lifetime. Those affirming this were identified as having a smoking history, whereas participants who reported a cigarette consumption of less than 100 were categorized as non-smokers.

### 2.5 Data analysis

All statistical analyses were conducted utilizing DecisionLinnc 1.0 software. Categorical variables are detailed through both the frequency counts and the corresponding weighted percentages. The use of weights in our analysis accounts for the complex sampling design of NHANES, ensuring that our estimates are representative of the U.S. population. A p-value of less than 0.05 was established as the threshold for statistical significance. Chi-square tests, univariate and multivariate logistic regression analysis were performed test the association of employment with depression. After accounting for various covariates, we finally developed a suite of five logistic regression models, leveraging weighted data to delve into the relationship between employment and depression. Model I: adjusted for non. Model II: adjusted for age, gender, race, marital status, education level, PIR, BMI, diabetes, hypertension, smoke status and alcohol status. Model III: adjusted for gender, race, marital status, education level, PIR, BMI, diabetes, hypertension, smoke status and alcohol status. Model IV: adjusted for age, gender, race, education level, PIR, BMI, diabetes, hypertension, smoke status and alcohol status. Model V: adjusted for age, gender, race, marital status, education level, BMI, diabetes, hypertension, smoke status and alcohol status. What’s more, we conducted subgroup analyses by segmenting data across age, gender, marital status, and PIR, then applied logistic regression and interaction tests to identify any notable trends within these groups.

## 3 Results

**Figure 1:**
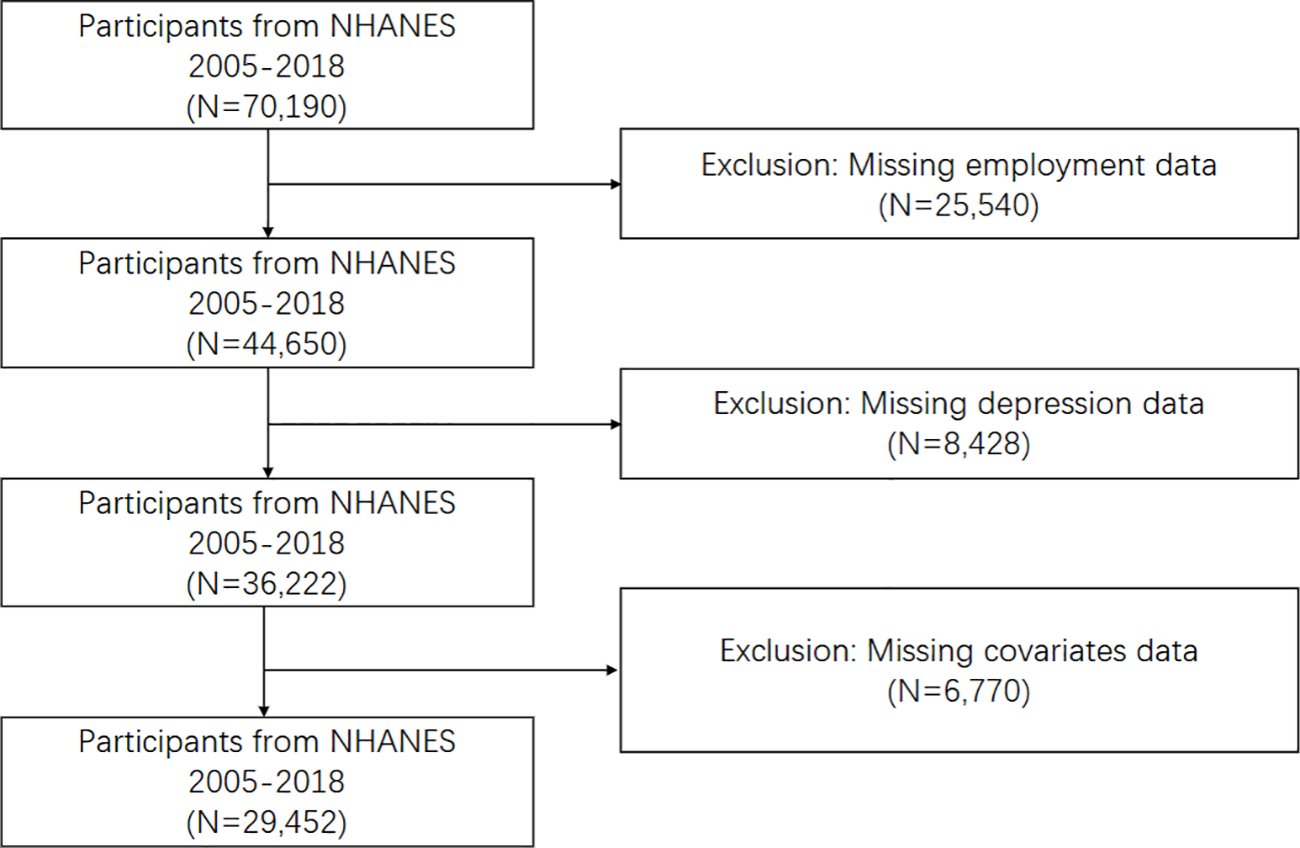
The flow chart of participant screening. NHANES, National Health and Nutrition Examination Survey.

Of 70190 participants, 25540 were excluded due to incomplete employment data, and an additional 8,428 were omitted because of missing depression data. Finally, 29452 participants with full information were enrolled in our study (as depicted in **Figure 1**). Approximately 180 million adults were identified by weighing the samples.

### 3.1 Baseline characteristics according to depression status

We identified significant associations between various sociodemographic and health characteristics and the presence of depression (**Table 1**). The overall prevalence of depression was 8.61%. Notably, middle aged individuals (40-59 years) were more likely to report depression. Females exhibited a higher rate of depression than males. Ethnic disparities were observed, with Non-Hispanic Black and other Hispanic individuals having higher depression rates (13.42% and 7.05%, respectively). Marital status also played a role, with those never married or widowed/divorced/separated showing higher depression rates. Education level was inversely related to depression, with those having less than a high school education reporting higher rates (24.97%) than those with college education or higher (48.18%). Economic status, as indicated by the PIR, was significantly linked to depression, with those in the lowest PIR category having a higher rate (41.67%) than those in the highest (22.03%). Health factors such as obesity, diabetes, hypertension, and smoking were also significantly associated with depression. What’s more, employment status showed a clear trend, with non-employed individuals having the highest rate of depression, while full-time workers had the lowest. These findings underscore the multifaceted determinants of depression and highlight the need for targeted interventions across various societal strata.

**Table 1:** Characteristics of participants stratified by depression category (NHANES 2005–2018).

Characteristic	Overall N(weighted%)	Depression N(weighted%)		*p*-value
		No	Yes	
Number	29452	26917(91.39)	2535(8.61)	
Age 20-39 40-59 ≥60	10125(36.87) 9635(37.62) 9692(25.51)	9315(36.96) 8631(37.22) 8971(25.83)	810(35.82) 1004(42.55) 721(21.63)	<0.001
Gender Female Male	14972(51.14) 14480(48.86)	13342(50.03) 13575(49.97)	1630(64.80) 905(35.20)	<0.001
Race Mexican American Other Hispanic Non-Hispanic White Non-Hispanic Black Other Race	4454(8.00) 2637(5.10) 13096(69.14) 6242(10.73) 3023(7.02)	4076(8.03) 2337(4.95) 11986(69.49) 5682(10.51) 2836(7.02)	378(7.60) 300(7.05) 1110(64.85) 560(13.42) 187(7.07)	<0.001
Marital status Married/Living with partner Never married Widowed/divorced/separated	11851(38.52) 15353(55.97) 2248(5.51)	10432(37.10) 14474(57.56) 2011(5.33)	1419(55.87) 879(36.39) 237(7.74)	<0.001
Education level <High school =High school ≥College	6807(15.36) 6827(22.82) 15818(61.82)	5956(14.58) 6188(22.49) 14773(62.93)	851(24.97) 639(26.84) 1045(48.18)	<0.001
PIR <1.3 ≥1.3, <3.5 ≥3.5	9114(20.69) 11175(35.72) 9163(43.59)	7770(18.98) 10333(35.67) 8814(45.35)	1344(41.67) 842(36.30) 349(22.03)	<0.001
BMI (kg/m^2^) <18.5 ≥18.5, <24 ≥24	446(1.51) 6139(21.79) 22867(76.70)	397(1.48) 5692(22.02) 20828(76.50)	49(1.81) 447(18.97) 2039(79.22)	0.016
Diabetes No Yes	25592(90.25) 3860(9.75)	23575(90.77) 3342(9.23)	2017(83.86) 518(16.14)	<0.001
Hypertension No Yes	19014(68.48) 10438(31.52)	17668(69.45) 9249(30.55)	1346(56.54) 1189(43.46)	<0.001
Smoke status No Yes	16107(54.77) 13345(45.23)	15097(56.11) 11820(43.89)	1010(38.31) 1525(61.69)	<0.001
Alcohol status No Yes	7497(20.17) 21955(79.83)	6878(20.17) 20039(79.83)	619(20.12) 1916(79.88)	0.958
Employment Non-employed Part time Full time	13292(37.55) 3818(13.61) 12342(48.85)	11600(35.82) 3559(13.74) 11758(50.44)	1692(58.76) 259(11.90) 584(29.34)	<0.001

BMI, body mass index; PIR, poverty-to-income ratio; NHANES, national health and Nutrition Examination Survey.

### 3.2 Baseline characteristics according to employment status

**Table 2** displays the basic characteristics of participants categorized by employment status, showing that employment status was significantly associated with all variables. The distribution revealed 45.13% non-employed, 12.96% part-time, and 41.91% full-time workers. As working hours increased, the proportion of women decreased. On the contrary, the prevalence of depression significantly reduced (non-employed: 11.77%, part time: 6.58%, full time: 4.52%). Besides, full-time workers were more inclined to exhibit married/living with partner, higher education level, higher PIR values and alcohol consumption. In addition, individuals with less working time were more likely to experience diabetes, hypertension and smoking.

**Table 2:** Characteristics of participants stratified by Employment category (NHANES 2005–2018).

Characteristic	Overall N(weighted%)	Employment status N(weighted%)			*p*-value
		Non-employed	Part time	Full time	
Number	29452	13292(45.13)	3818(12.96)	12342(41.91)	
Age 20-39 40-59 ≥60	10125(36.87) 9635(37.62) 9692(25.51)	2985(24.82) 2995(26.08) 7312(49.09)	1716(45.04) 1191(35.05) 911(19.91)	5424(43.86) 5449(47.19) 1469(8.94)	<0.001
Gender Female Male	14972(51.14) 14480(48.86)	7499(60.06) 5793(39.94)	2202(59.49) 1616(40.51)	5271(41.97) 7071(58.03)	<0.001
Race Mexican American Other Hispanic Non-Hispanic White Non-Hispanic Black Other Race	4454(8.00) 2637(5.10) 13096(69.14) 6242(10.73) 3023(7.02)	1765(6.75) 1142(4.75) 6363(70.53) 2857(11.45) 1165(6.52)	580(8.35) 329(4.98) 1697(68.20) 780(10.85) 432(7.62)	2109(8.86) 1166(5.41) 5036(68.34) 2605(10.15) 1426(7.24)	<0.001
Marital status Married/Living with partner Never married Widowed/divorced/separated	11851(38.52) 15353(55.97) 2248(5.51)	4854(34.40) 6564(53.89) 1874(11.72)	1797(43.84) 1856(53.02) 165(3.14)	5200(40.20) 6933(58.39) 209(1.41)	<0.001
Education level <High school =High school ≥College	6807(15.36) 6827(22.82) 15818(61.82)	3738(19.89) 3293(25.07) 6261(55.05)	773(13.69) 843(20.73) 2202(65.58)	2296(12.35) 2691(21.67) 7355(65.97)	<0.001
PIR <1.3 ≥1.3, <3.5 ≥3.5	9114(20.69) 11175(35.72) 9163(43.59)	5412(30.19) 4971(37.77) 2909(32.04)	1295(24.26) 1439(35.12) 1084(40.62)	2407(12.38) 4765(34.32) 5170(53.30)	<0.001
BMI (kg/m^2^) <18.5 ≥18.5, <24 ≥24	446(1.51) 6139(21.79) 22867(76.70)	248(2.03) 2659(21.16) 10385(76.81)	66(1.85) 986(27.18) 2766(70.98)	132(1.01) 2494(20.78) 9716(78.21)	<0.001
Diabetes No Yes	25592(90.25) 3860(9.75)	10718(84.75) 2574(15.25)	3469(93.29) 349(6.71)	11405(93.63) 937(6.37)	<0.001
Hypertension No Yes	19014(68.48) 10438(31.52)	6968(56.23) 6324(43.77)	2766(75.07) 1052(24.93)	9280(76.05) 3062(23.95)	<0.001
Smoke status No Yes	16107(54.77) 13345(45.23)	6572(49.59) 6720(50.41)	2222(58.54) 1596(41.46)	7313(57.71) 5029(42.29)	<0.001
Alcohol status No Yes	7497(20.17) 21955(79.83)	4045(25.99) 9247(74.01)	907(18.56) 2911(81.44)	2545(16.14) 9797(83.86)	<0.001
Depression No Yes	26917(92.48) 2535(7.52)	11600(88.23) 1692(11.77)	3559(93.42) 259(6.58)	11758(95.48) 584(4.52)	<0.001

BMI, body mass index; PIR, poverty-to-income ratio; NHANES, national health and Nutrition Examination Survey.

### 3.3 Association between employment and depression stratified by gender

The overall analysis demonstrated significant associations between employment and depression. Compared to the non-employed, both part-time workers (with an odds ratio [OR] of 0.53, 95% confidence interval [CI] 0.44 to 0.63) and full-time workers (OR 0.35, 95% CI 0.31 to 0.40) had reduced odds of depression, with both P-values being less than 0.001. Stratified by gender, the relationship remained significant for both females and males (**Table 3**). Female part-time workers had an OR of 0.51 (95% CI 0.41 to 0.63) and full-time workers an OR of 0.44 (95% CI 0.38 to 0.52). Similarly, for males, part-time workers had an OR of 0.57 (95% CI 0.42 to 0.78) and full-time workers an OR of 0.32 (95% CI 0.26 to 0.39). These findings held true after adjusting for various covariates, including age, race, marital status, education level, PIR, BMI, diabetes, hypertension, smoking status, and alcohol status.

**Table 3:** Weighted univariate and multivariable logistic regression analysis between employment and depression, gender stratification (NHANES 2005–2018).

Gender	Employment status	Depression status	
		Model I	Model II
		OR (95% CI), *P*	OR (95% CI), *P*
Overall	Non-employed	Reference	Reference
	Part time	0.53(0.44, 0.63), 0.000	0.53(0.44, 0.64), 0.000
	Full time	0.35(0.31, 0.40), 0.000	0.42(0.36, 0.48), 0.000
Female	Non-employed	Reference	Reference
	Part time	0.51(0.41, 0.63), 0.000	0.51(0.40, 0.64), 0.000
	Full time	0.44(0.38, 0.52), 0.000	0.46(0.38, 0.55), 0.000
Male	Non-employed	Reference	Reference
	Part time	0.57(0.42, 0.78), 0.000	0.57(0.42, 0.79), 0.001
	Full time	0.32(0.26, 0.39), 0.000	0.36(0.28, 0.46), 0.000

Model I: adjusted for non. Model II: adjusted for age, gender, race, marital status, education level, PIR, BMI, diabetes, hypertension, smoke status and alcohol status. For the models in which the analysis was stratified by gender, gender was not included as a covariate. *p*< 0.05 presents significant difference. BMI, body mass index; PIR, poverty-to-income ratio; CI, confidence interval; OR, odds ratio.

### 3.4 Association between employment and depression stratified by age

Moving forward, we conducted stratified logistic regression analyses segmented by age. As depicted in **Table 4**, Across all age groups, individuals employed part-time or full-time demonstrated lower odds of depression compared to their non-employed counterparts. For those aged 20-39, the odds of depression were substantially reduced for part-time (OR 0.54, 95% CI 0.42-0.70) and full-time workers (OR 0.35, 95% CI 0.29-0.43). This trend persisted in the 40-59 age bracket, with part-time workers showing an OR of 0.34 (95% CI 0.25-0.46) and full-time workers an OR of 0.19 (95% CI 0.16-0.24). Among individuals aged 60 and above, part-time employment was associated with an OR of 0.45 (95% CI 0.27-0.75), while full-time employment had an OR of 0.46 (95% CI 0.30-0.70) for depression. After adjusting for all other variables in Model III, the associations remained robust for the younger age groups but showed less pronounced effects for the oldest age group. Specifically, the adjusted odds for full-time employment in individuals aged 60 and above increased to an OR of 0.73 (95% CI 0.47-1.13), suggesting a weaker association in this demographic.

**Table 4:** Weighted univariate and multivariable logistic regression analysis between employment and depression, age stratification (NHANES 2005–2018).

Age	Employment status	Depression status	
		Model I	Model III
		OR (95% CI), *P*	OR (95% CI), *P*
20-39	Non-employed	Reference	Reference
	Part time	0.54(0.42, 0.70), 0.000	0.62(0.48, 0.81), 0.000
	Full time	0.35(0.29, 0.43), 0.000	0.51(0.41, 0.63), 0.000
40-59	Non-employed	Reference	Reference
	Part time	0.34(0.25, 0.46), 0.000	0.43(0.31, 0.58), 0.000
	Full time	0.19(0.16, 0.24), 0.000	0.31(0.25, 0.38), 0.000
≥60	Non-employed	Reference	Reference
	Part time	0.45(0.27, 0.75), 0.002	0.58(0.34, 0.98), 0.044
	Full time	0.46(0.30, 0.70), 0.000	0.73(0.47, 1.13), 0.159

Model I: adjusted for non. Model III: adjusted for gender, race, marital status, education level, PIR, BMI, diabetes, hypertension, smoke status and alcohol status. *p*< 0.05 presents significant difference. BMI, body mass index; PIR, poverty-to-income ratio; CI, confidence interval; OR, odds ratio.

### 3.5 Association between employment and depression stratified by marital status

The relationship between marital status, employment status, and depression status was examined using two distinct models. Model I served as a baseline, while Model IV incorporated a broader range of confounding factors. As shown in **Table 5**, among those married or living with a partner, both part-time (OR 0.44, 95% CI 0.35-0.56) and full-time employment (OR 0.34, 95% CI 0.29-0.40) were significantly associated with lower odds of depression compared to non-employment. After adjusting for additional variables in Model IV, the odds for part-time employment increased slightly (OR 0.52, 95% CI 0.41-0.66), but remained significant, as did full-time employment (OR 0.41, 95% CI 0.34-0.48). For never-married individuals, the odds of depression were also lower for those working part-time (OR 0.56, 95% CI 0.41-0.75) and full-time (OR 0.33, 95% CI 0.27-0.41). These associations persisted in Model IV with adjusted odds for part-time employment increasing to an OR of 0.66 (95% CI 0.49-0.90) and full-time to an OR of 0.45 (95% CI 0.36-0.56). However, for those widowed, divorced, or separated, the associations were not statistically significant. Part-time employment showed an OR of 0.70 (95% CI 0.30-1.67) in Model I, which increased to 0.80 (95% CI 0.33-1.91) in Model IV, while full-time employment showed an OR of 0.78 (95% CI 0.36-1.68) in Model I, increasing to 0.98 (95% CI 0.45-2.10) in Model IV.

**Table 5:** Weighted univariate and multivariable logistic regression analysis between employment and depression, marital status stratification (NHANES 2005–2018).

Marital status	Employment status	Depression status	
		Model I	Model IV
		OR (95% CI), *P*	OR (95% CI), *P*
Married/Living with partner	Non-employed	Reference	Reference
	Part time	0.44(0.35, 0.56), 0.000	0.52(0.41, 0.66), 0.000
	Full time	0.34(0.29, 0.40), 0.000	0.41(0.34, 0.48), 0.000
Never married	Non-employed	Reference	Reference
	Part time	0.56(0.41, 0.75), 0.000	0.66(0.49, 0.90), 0.009
	Full time	0.33(0.27, 0.41), 0.000	0.45(0.36, 0.56), 0.000
Widowed/divorced/separated	Non-employed	Reference	Reference
	Part time	0.70(0.30, 1.67), 0.425	0.80(0.33, 1.91), 0.612
	Full time	0.78(0.36, 1.68), 0.531	0.98(0.45, 2.10), 0.950

Model I: adjusted for non. Model IV: adjusted for age, gender, race, education level, PIR, BMI, diabetes, hypertension, smoke status and alcohol status. *p*< 0.05 presents significant difference. BMI, body mass index; PIR, poverty-to-income ratio; CI, confidence interval; OR, odds ratio.

### 3.6 Association between employment and depression stratified by PIR

In the analysis of the relationship between PIR, employment status, and depression status, significant findings emerged (as shown in **Table 6**). For individuals with a PIR less than 1.3, part-time employment showed an OR of 0.50 (95% CI 0.39 to 0.63), and full-time employment an OR of 0.38 (95% CI 0.32 to 0.47) for depression, both significantly less than the reference group of non-employed. These ORs slightly increased but remained significant in Model V after adjusting for various covariates, to 0.55 (95% CI 0.43 to 0.71) and 0.51(95% CI 0.41 to 0.62), respectively. In the PIR range of 1.3 to 3.5, part-time and full-time employments were also associated with reduced odds of depression, with ORs of 0.58 (95% CI 0.43 to 0.78) and 0.40 (95% CI 0.32 to 0.49) in Model I, and 0.58 (95% CI 0.42 to 0.79) and 0.42 (95% CI 0.33 to 0.53) in Model V. However, for those with a PIR of 3.5 or higher, the association was not statistically significant, with ORs for part-time and full-time employments being 0.77 and 0.69 in Model I, and 0.83 and 0.75 in Model V. These results indicate that employment is linked to decreased odds of depression, especially in individuals with lower PIR, suggesting a complex interaction between economic status, employment, and mental health.

**Table 6:** Weighted univariate and multivariable logistic regression analysis between employment and depression, PIR stratification (NHANES 2005–2018).

PIR	Employment status	Depression status	
		Model I	Model V
		OR (95% CI), *P*	OR (95% CI), *P*
<1.3	Non-employed	Reference	Reference
	Part time	0.50(0.39, 0.63), 0.000	0.55(0.43, 0.71), 0.000
	Full time	0.38(0.32, 0.47), 0.000	0.51(0.41, 0.62), 0.000
≥1.3, <3.5	Non-employed	Reference	Reference
	Part time	0.58(0.43, 0.78), 0.000	0.58(0.42, 0.79), 0.001
	Full time	0.40(0.32, 0.49), 0.000	0.42(0.33, 0.53), 0.000
≥3.5	Non-employed	Reference	Reference
	Part time	0.77(0.50, 1.20), 0.246	0.83(0.53, 1.31), 0.423
	Full time	0.69(0.52, 0.92), 0.012	0.75(0.55, 1.02), 0.064

Model I: adjusted for non. Model V: adjusted for age, gender, race, marital status, education level, BMI, diabetes, hypertension, smoke status and alcohol status. *p*< 0.05 presents significant difference. BMI, body mass index; PIR, poverty-to-income ratio; CI, confidence interval; OR, odds ratio.

### 3.7 Subgroup interaction analysis

**Figure 2:**
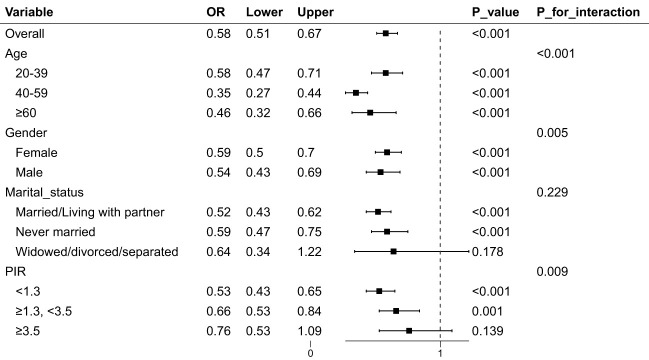
The association between employment and depression by different subgroups. PIR, poverty-to-income ratio; OR, odds ratio.

Finally, we conducted subgroup analysis to determine whether employment has similar depressive effects among different populations. As shown in **Figure 2**, the findings revealed that while there is a significant correlation between employment status and the presence of depressive symptoms across various subgroups defined by age, gender, marital status, and PIR, this correlation is not significant when the poverty-to-income ratio (PIR) is 3.5 or higher or when marital status is categorized as widowed/divorced/separated (p > 0.05).” Besides, significant interactions were found in age, gender and PIR (p < 0.05).

## 4 Discussion

The current study, leveraging a nationally representative sample from NHANES, provides compelling evidence of the intricate relationship between employment status, sociodemographic factors, and depression. Our findings underscore the multifaceted nature of depression, highlighting the critical role of employment as a social determinant of mental health. The findings from this study offer valuable insights into the multifaceted determinants of depression, echoing previous research that has underscored the complexity of this mental health condition (12).

The higher prevalence of depression among females and certain ethnic groups, such as Non-Hispanic Black and other Hispanic individuals, is a well-documented phenomenon (13, 14). These disparities may be attributed to a range of factors, including social determinants of health, cultural factors, and differential access to mental health care. The inverse relationship between education level and depression is supported by a substantial body of research, which has consistently shown that higher levels of education are protective against depression (15, 16). This may be due to increased social support, greater access to resources, and higher socioeconomic status associated with higher education. The link between economic status, as indicated by the PIR, and depression aligns with the principle that financial strain is a significant risk factor for mental health problems (17). The observed gradient in depression prevalence across different PIR categories highlights the importance of economic stability for mental well-being.

The association between employment status and depression is a complex one and has been extensively studied. Employment is not only a determinant of economic stability but also a source of social identity and a provider of social support, which can be protective against depression (4). Our findings that non-employed individuals have a higher rate of depression compared to part-time and full-time workers are consistent with existing literature that highlights the protective role of employment in mental health (18). Stratified by gender, the results showed a consistent trend, with females and males in part-time and full-time employment having lower odds of depression compared to the non-employed. The gender-specific ORs suggest that while women may have a higher prevalence of depression, the beneficial effect of employment on mental health is observed in both genders. Unlike our research, Jae et al. suggests that unstable and long-term work among South Korean females does not have an impact on the incidence of depression (18). This prompts that the relationship between employment characteristics and depression may vary significantly across different cultural contexts and societal structures. This discrepancy could be attributed to several factors, including cultural attitudes towards work, the social perception of women in the workforce, and the specific economic conditions prevalent in South Korea. It also underscores the importance of localized research to tailor mental health interventions and public health policies that are responsive to the unique needs and circumstances of different populations.

The relationship between age, employment status, and depression status further nuances our understanding. Notably, the odds of depression were lower for employed individuals across all age groups, with the most pronounced effect observed in the 40-59 age group. This age bracket typically represents individuals in the prime of their careers, with established work roles and social networks that contribute to a sense of purpose and belonging. Employment during these middle-aged years often comes with increased job stability, higher income, and greater social support, all of which are known to positively influence mental health (19). The stability of employment in the 40-59 age group may also provide a buffer against the psychological stressors that can accompany the challenges of middle age, such as caring for aging parents and guiding children through their formative years. This period, sometimes referred to as the “sandwich generation,” can be particularly stressful, and the stability that full-time employment offers may help mitigate the impact of these stressors on mental health. In contrast, the less pronounced effect of employment on depression in the oldest age group (≥60) is likely influenced by a variety of factors unique to this demographic. For some, continued employment may provide a sense of purpose and social engagement, while for others, retirement may offer a welcome respite and a chance to pursue personally meaningful activities. The attenuated effect of employment on depression in the oldest age group may also reflect cohort effects, with older individuals having different attitudes towards work and retirement compared to younger generations (20, 21). Another possibility is that the proportion of retirees in the non-employed population has sharply increased among those over 60 years old, and these elderly people who enjoy retirement benefits have reduced the depression baseline of the non-employed population. Overall, the relationship between age, employment status, and depression is complex and influenced by a range of social, economic, and individual factors. The findings from this study highlight the importance of considering life stage and the unique circumstances of different age groups when developing mental health policies and interventions. For middle-aged adults, promoting stable employment opportunities and workplace mental health initiatives can be a key strategy for preventing depression. For older adults, it is crucial to support the transition to retirement, foster social engagement, and address the broader social determinants of mental health.

Marital status emerged as another significant factor in the context of employment and depression. The findings from this study indicate that individuals who are married or living with a partner and are employed, either part-time or full-time, exhibit lower odds of depression. This finding is in line with research suggesting that marital status can influence mental health, possibly due to the social and emotional support provided by a partner (22). The association between marital status, employment, and depression is a complex interplay of social dynamics that can significantly influence mental health outcomes. Marriage and cohabitation often involve shared responsibilities, companionship, and a sense of belonging, which can contribute to an individual’s overall well-being and resilience against stressors. The social integration that comes with being in a relationship can also lead to increased social interaction and a more extensive support network, which are both protective factors for mental health. In contrast, the higher odds of depression observed among the never-married group may be linked to the lack of a partner’s support system. Never-married individuals may face unique challenges, such as social isolation or economic instability, which can increase vulnerability to mental health problems (23). Societal norms and expectations surrounding marriage can lead to perceived social pressure and stigma for those who remain unmarried, potentially contributing to feelings of loneliness or depression. What’s more, when analyzing the relationship between the employment status of widowed, divorced, or separated individuals and depression, our research results indicate that compared to non-employed status, the correlation between part-time or full-time work and depression is not statistically significant. This may indicate that in this specific group, the impact of employment status on depression is limited, or there are other more complex factors affecting their mental health. Individuals who are widowed, divorced, or separated may have experienced significant life changes and emotional challenges, which may have a more direct impact on their mental health than their employment status. For example, losing a partner may lead to profound grief and loneliness, while divorce may be accompanied by economic and emotional uncertainty. These experiences may lead to an increase in stress levels, which in turn increases the risk of depression. In addition, individuals in this group may face the challenge of redefining their self-identity and social roles, which may affect their social support network and the stability of their daily lives. It is important to note, however, that the relationship between marital status and depression is not unidirectional. While being married or in a partnership can provide support and reduce depression risk, depression can also impact relationship stability and satisfaction (24). Marital status not only affects the onset of depression, but also affects the prognosis of depression. A study from China has shown that the prognosis of single or unmarried patients may be slightly worse than that of married patients (25). Our study emphasizes the importance of considering both marital status and employment status when assessing depression risk.

The examination of the PIR in relation to employment and depression provides critical insights into the economic determinants of mental health. The findings from this study suggest that employment plays a significant role in reducing the odds of depression, particularly among individuals with a lower PIR. This association underscores the multifaceted benefits of employment that extend beyond income generation. For those with a lower PIR, employment is not only a source of income but also a means to social integration, routine, and a sense of purpose. These psychosocial factors are integral to mental well-being and can be particularly protective for those who might otherwise be facing economic hardship and the stressors associated with poverty (26). Employment can also provide access to resources such as health insurance and employee assistance programs, which can be crucial for the early detection and management of mental health issues. The less pronounced association between employment and depression at higher PIR levels may reflect the fact that financial stability can buffer against the stressors that can lead to mental health problems. Individuals with higher PIRs may have sufficient economic resources to manage life’s challenges without the same level of dependence on the protective factors associated with employment. This is not to say that employment is not beneficial for those with higher PIRs, but rather that the mental health benefits of employment may be more critical for those with fewer economic resources. It is also important to consider that higher PIR levels may be indicative of other factors that influence mental health, such as higher education levels, better job quality, and greater social support networks. These factors can contribute to resilience and may alter the relationship between employment and mental health in a way that is not as pronounced as it is for those with lower PIRs. These results highlight the need for policies and interventions that promote employment opportunities, particularly for those in lower income brackets. This includes initiatives that provide job training, education, and support for individuals living in poverty.

While the current study offers valuable insights into the relationship between employment status, sociodemographic factors, and depression, it is not without limitations. The cross-sectional design of the study precludes any causal inferences, meaning that the directionality of the observed associations cannot be determined. Additionally, the reliance on self-reported data for both employment status and depression may introduce reporting biases, potentially affecting the accuracy of the results. The generalizability of the findings may also be limited by the specific demographic included in the NHANES dataset, and the results may not be fully representative of all populations. Furthermore, the study did not account for certain potential confounders, such as individual personality traits, work-related stressors, or the quality of marital relationships, which could influence both employment status and depression. Moreover, due to the existing questionnaire design of NHANES, we could only define employment based on the respondents’ employment status from the last week, which is a less rigorous approach. Future research employing longitudinal designs and objective mental health assessments could provide a clearer understanding of the causal pathways linking employment, socioeconomic status, and depression. Incorporating measures of work environment, job satisfaction, and social support at the workplace could also offer a more nuanced perspective on how employment influences mental health. Despite these limitations, the study contributes meaningfully to the existing literature and provides a foundation for further investigation into the complex interplay between employment, sociodemographic factors, and depression.

## 5 Conclusion

In conclusion, the study offers a profound analysis of the relationship between employment and depression, revealing that employment serves as a multifaceted determinant of mental health. Employment’s significance extends beyond economic stability, providing social identity, support, structure, and a sense of purpose, all of which are instrumental in protecting against depression. The study’s findings consistently show that individuals in part-time and full-time employment, particularly in the middle age group and those with lower PIRs, have reduced odds of depression, underscoring the protective role of employment. Additionally, the study highlights the importance of marital status and its interaction with employment in influencing mental health outcomes. These findings underscore the necessity for policies that promote stable and fulfilling employment opportunities as a critical strategy for depression prevention, especially for vulnerable populations. The research also calls for workplace mental health initiatives that can further support employees’ well-being. By considering the complex interplay between employment, sociodemographic factors, and mental health, this study contributes valuable insights for policymakers, mental health professionals, and community leaders in their efforts to address and alleviate depression.

## Data Availability

The original contributions presented in the study are included in the article. Further inquiries can be directed to the corresponding author.
